# Lysosome-directed targeted protein degradation technologies for overcoming cancer drug resistance: mechanisms, design principles, and therapeutic opportunities

**DOI:** 10.1080/10717544.2026.2679844

**Published:** 2026-05-27

**Authors:** Huixin Mao, Ying Zhang, Mingqi Liu, Yao Yao, Yujie Peng, Xuwei Zhou, Hao Huang, Weidong Fei, Caihong Zheng, Yiqing Ye

**Affiliations:** a Research Center for Clinical Pharmacy, College of Pharmaceutical Sciences, Zhejiang University, Hangzhou, China; b Women’s Hospital, Zhejiang University School of Medicine, Hangzhou, China; c Zhejiang Key Laboratory of Maternal and Infant Health, Hangzhou, China

**Keywords:** LYTACs, lysosome, tumor, drug resistance, degradation

## Abstract

Targeted protein degradation (TPD) has emerged as a promising therapeutic strategy to address cancer drug resistance by enabling the selective and efficient degradation of disease-associated proteins through cellular mechanisms. Since 2020, lysosome-targeting chimeras (LYTACs) have gained attention for expanding targeted protein degradation to extracellular and membrane-associated disease-related proteins beyond the reach of proteolysis-targeting chimeras (PROTACs). In practice, LYTACs can overcome resistance by degrading instead of inhibiting them. This efficient and durable removal of resistance-associated drivers thereby suppresses compensatory signaling, restores drug sensitivity, and contributes to remodeling the tumor microenvironment. To facilitate the clinical translation of this emerging technology, this review systematically compares the mechanistic and functional advantages of LYTACs over PROTACs, summarizes key design principles, and categorizes major LYTAC modalities, including AbTACs, MoDE-As, and nano-LYTACs. We further discuss their *in vivo* pharmacological behaviors, with particular emphasis on stability, tumor selectivity, and degradation efficiency. Importantly, this article comprehensively highlights recent advances in the application of LYTACs for overcoming therapeutic resistance, including resistance to chemotherapy, targeted therapy, and immunotherapy. Finally, current translational bottlenecks, such as delivery efficiency, receptor heterogeneity, and pharmacokinetic limitations, are critically analyzed. Collectively, while still in its early stages of development, LYTAC-based lysosomal degradation represents a promising, paradigm-shifting strategy for targeting previously intractable mechanisms of cancer resistance.

## 1. Introduction

Although substantial advances have been achieved in cancer therapy, including cytotoxic chemotherapy, molecularly targeted agents, and immune checkpoint blockade, the emergence of drug resistance remains a pervasive barrier to durable clinical benefit (Li et al. 2025a). For example, in patients with estrogen receptor (ER)-positive, human epidermal growth factor receptor 2 (HER2)-negative breast cancer, approximately 30% develop resistance to endocrine therapy within 5 years, ultimately leading to disease recurrence (Schagerholm Stanev et al. 2025). Mechanistically, resistance to conventional small-molecule inhibitors is frequently driven by target mutations that impair drug binding, gene amplification, compensatory pathway activation, and increased drug efflux, all of which arise under sustained pharmacological pressure from occupancy-driven inhibition.

**Figure 1.**
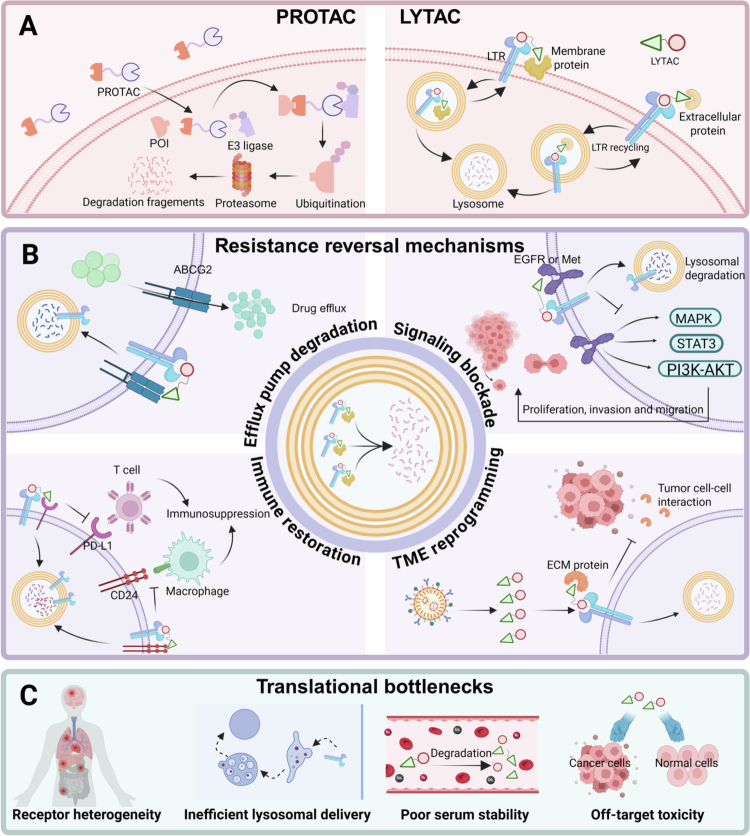
(A) The mechanism of action of PROTACs and LYTACs. (B) Degradation of proteins by LYTAC technology overcomes drug resistance. (C) Major limitations for LYTACs clinical translation. Created in BioRender. https://BioRender.com/zfrd6jb.

Targeted protein degradation (TPD) has emerged as an alternative therapeutic paradigm that eliminates disease-causing proteins rather than transiently inhibiting their activity. PROTACs are heterobifunctional molecules composed of a ligand for the protein of interest (POI), a ligand for an E3 ubiquitin ligase, and a chemical linker connecting the two (Figure 1A). By simultaneously engaging the POI and an E3 ligase, PROTACs induce the formation of a ternary complex that promotes polyubiquitination of the target protein and its subsequent degradation by the 26S proteasome (Burke et al. 2022). This event-driven, catalytic mechanism enables PROTACs to overcome key limitations of traditional inhibitors, including the ability to target proteins lacking enzymatic activity. However, because PROTACs rely on the intracellular ubiquitin–proteasome system (UPS), their applicability is largely restricted to intracellular proteins and is less effective against extracellular or membrane-associated targets (Zhang et al. 2019).

To address this limitation, lysosome-targeting chimeras (LYTACs) have been developed to extend targeted protein degradation to extracellular and membrane proteins (Banik et al. 2020). LYTACs are bifunctional constructs that bind both a target protein and lysosomal trafficking receptors (LTRs), a class of transmembrane receptors responsible for shuttling glycosylated cargo from the cell surface or Golgi apparatus to lysosomes for degradation (Figure 1A). Representative LTRs include the cation-independent mannose-6-phosphate receptor (CI-M6PR), which recognizes mannose-6-phosphate-modified ligands and mediates their endocytosis and lysosomal delivery. Upon simultaneous binding of the target protein and an LTR, LYTACs induce the formation of a ternary complex that undergoes clathrin-mediated endocytosis (Yang et al. 2023). Following internalization, LTRs dissociate within early endosomes and recycle back to the plasma membrane, while the LYTAC target complex is trafficked to lysosomes, where the target protein is degraded by lysosomal hydrolases.

From a therapeutic perspective, the selection of appropriate targets is central to LYTAC design. Many key mediators of drug resistance are extracellular or membrane proteins whose functions are governed by their extracellular domains. These include immune checkpoint ligands such as programmed death-ligand 1 (PD-L1), receptor tyrosine kinases, such as epidermal growth factor receptor (EGFR) and other surface proteins involved in tumor microenvironment interactions (Liu et al. 2023; Simon and Fitzgerald 2016). By enabling the degradation, not merely inhibition of these proteins, LYTACs can simultaneously abrogate oncogenic signaling, disrupt immune evasion, and remodel tumor stromal crosstalk (Figure 1B). Consistent with this mechanism, the degradation of PD-L1 has been shown to achieve more than 90% target reduction, leading to enhanced antitumor immunity, significant tumor regression in preclinical models, and prolonged survival (Xiao et al. 2025c).

In general, emerging LYTACs have significantly improved the efficiency of target protein degradation through the design of novel ligands and the identification of new lysosome-targeting receptors. In this review, we aim to delineate the molecular and cellular mechanisms underlying LYTAC-mediated degradation, summarize current design strategies with an emphasis on ligand selection, linker engineering, and target specificity (as summarized in Table 1), and critically evaluate their preclinical therapeutic applications in the context of drug-resistant malignancies. By explicitly connecting mechanistic insights with design logic and therapeutic outcomes, this article seeks to provide a coherent conceptual framework that both complements and extends previously published LYTAC-focused reviews, with particular emphasis on translational considerations and resistance reversal strategies (Figure 1C).

**Table 1.** Summary of LYTAC-based degradation technologies for overcoming cancer drug resistance.

LYTACs	POI/POI ligand	LTR/LTR ligand	Linker/Scaffold	Experimental model	References
NLTC	PD-L1/Acryloylated BMS-8	CI- M6PR/Acryloylated M6P	PH-responsive detach able PEG shell	B16F10 tumor bearing C57BL/6 mice	Xing et al. (2025)
Pep-TAC	PD-L1/k-ASF-embedded covalent peptide	TFRC/Peptide DT7	Direct peptide bond linkage	MC38 tumor bearing mice, intravenous injection (1.3 mg/kg, 3.9 mg/kg)	Xiao et al. (2025b)
Covalent LYTAC	PD-L1/DBCO-modified aptamer	CI-M6PR/DNA aptamer	Triazole linkage via click chemistry	B16F10 tumor bearing C57BL/6 mice, intravenous injection (2 nmol) or intrathecal injection (1 nmol)	Li et al. (2023)
FRTAC	PD-L1/Antibody	FRα/Polyvalent folates	Polyethylene glycol	BALB/c nude mice bearing H292 tumors, intravenous injection (20 mg/kg)	Xiao et al. (2025a)
Nano CLY	CTGF/Peptide	CI-M6PR/M6P_3_	PEG_3_	MDA-MB-231 tumor bearing mice, intravenous injection (10 mg/kg)	Lin et al. (2025)
AptLYTAC	PTK7 and Met/Aptamer	CI-M6PR/Poly mannose-6-phosphate (bpM6P) polymer	Biotin-streptavidin conjugation	MDA-MB-231 cells *in vitro*	Duan et al. (2024)
Multivalent DNA LYTAC	c-Met/Aaptamer	Scavenger receptors A1/PolyG sequence	DNA hybridization	Xenograft A549 tumor bearing mouse mode (3 nmol)	Chen et al. (2025b)
A nucleic acid-based LYTAC	VEGFR/Peptide	CI-M6PR/M6P	DNA hybridization	n-AMD model in mice	Huang et al. (2024)
HGTAC	EGFR/Amantadine (Ada) conjugated antibody	ASGPR/β-CD-tri-GalNAc	Supramolecular host-guest interaction (β-CD/Ada)	HepG2 xenograft mouse model, intratumoral injection (4 mg/kg)	Chen et al. (2025c)
iLYTAC	EGFR and HER/Antibody	IGF2R/IGF2 mutants with amino acid combinations	Antibody-IGF2 fusion	JIMT-1 cells implanted in nude mice, intraperitoneal injection (10 mg/kg)	Pan et al. (2025)
PT-LYTAC	PTK7/NIR photoactive aptamer Sgc8-PA	IGFIIR/Aptamer	DNA hybridization	Colorectal tumor xenograft model (40 μM)	Zhang et al. (2025a)
TDF-LYTAC	PDGF/Aptamer	IGFIIR/Aptamer	Tetrahedral DNA framework	MCF-7 cells *in vitro*	Ning et al. (2025)
MoDE-A	Cytokine macrophage migration inhibitory factor/Carboxylic acid-terminated MIF tautomerase inhibitor	ASGPR/Trivalent GalNAc	PEG	Nude mice, intraperitoneal injection (1 mg/kg)	Caianiello et al. (2021)
AbTAC	PD-L1/Antibody	RNF43/Anti RNF43 Fab fragment	/	Triple-negative breast cancer, non-small cell lung cancer and advanced bladder cancer models *in vitro*	Cotton et al. (2021)
Supra-LYTAC	PD-L1/Peptide	Carbonic anhydrase IX/Peptides of acetazolamine ligands bound to lysine residues	Supramolecular nanofibers formed by peptide co-assembly	4T1 xenograft model, intrathecal injection (2.5 mg/kg)	Kim et al. (2025)
GFLD	PD-L1/Antibody	Glucose transporter Glut1/Glycooligomer	DBCO-N3 click chemistry conjugation	MDA-MB-231 cells *in vitro*	Luo et al. (2024)
KineTAC	PD-L1/Antibody	CXCR7/Cytokine CXCL12	Bispecific antibody Fc scaffold	HeLa cells *in vitro*	Pance et al. (2023)

## 2. Drug resistance proteins

### 2.1. Extracellular proteins

Remodeling of the extracellular matrix (ECM) in the tumor microenvironment significantly impedes drug diffusion and enhances drug resistance through mechanisms such as increased interstitial pressure and the release of pro-survival signals (Mai et al. 2024). For instance, excessive ECM deposition leads to elevated interstitial fluid pressure and tumor hypoxia, both of which reduce the efficacy of conventional chemotherapy, radiotherapy, and immunotherapy. Moreover, certain ECM proteins (e.g. ECM1) have been reported to enhance EGFR/MAPK signaling and promote MUC1/HER3 expression, thereby conferring resistance to trastuzumab in HER2-positive breast cancer (Lee et al. 2014). In ovarian cancer, the secreted isoform ECM1a can induce a stem-like phenotype, resulting in resistance to platinum-based therapies (Yin et al. 2021). These observations collectively highlight the therapeutic potential of targeting ECM microenvironment interactions to enhance drug delivery and interrupt resistance-associated signaling. Clinically, strategies aimed at reducing stromal pressure to improve drug delivery have shown efficacy. For example, in patients with advanced pancreatic cancer, the combination of losartan to decrease tumor interstitial pressure significantly enhanced the penetration of chemotherapeutic agents and led to improved clinical outcomes (Boucher et al. 2023). Collectively, these findings highlight the therapeutic potential of targeting the ECM and its associated proteins to facilitate drug delivery and disrupt resistance signaling pathways.

Notably, many proteins involved in ECM remodeling are secreted or membrane-associated, making them, in principle, amenable to degradation via the LYTAC strategy. LYTACs utilize ligands that bind lysosome trafficking receptors (e.g. CI-M6PR) to direct extracellular targets into cells for lysosomal degradation, thereby eliminating resistance-associated extracellular molecules. For example, the first LYTAC designed by Banik et al. employed multivalent M6P ligands to engage CI-M6PR and successfully degraded a variety of extracellular and transmembrane proteins. Based on this concept, employing LYTACs to remove ECM related proteins or their ligands could weaken the stromal barrier and improve chemotherapeutic penetration and efficacy. However, the ECM is often composed of highly dense collagen networks and protein complexes, which may present a physical barrier to LYTAC penetration. For instance, collagen fiber crosslinking forms a stiff matrix that can hinder the accessibility of LYTAC molecules to their targets. Recent studies have attempted to degrade the collagen receptor DDR1 using LYTACs, thereby disrupting the collagen barrier and restoring immune cell infiltration, offering a promising strategy for overcoming collagen-mediated resistance.

### 2.2. Membrane proteins

Cell membrane proteins are key mediators of multidrug resistance (MDR) in tumors. These proteins typically possess extracellular domains that are readily accessible for LYTAC targeting. In tumor cells, ATP-binding cassette (ABC) transporters (such as P-gp/MDR1, BCRP/ABCG2, and MRP1) are often overexpressed and actively efflux drugs, reducing intracellular drug concentrations and leading to MDR (Sarwar et al. 2024). Clinical studies have proved that acute lymphoblastic leukemia patients with high expression of LAMP1 and P-gp proteins have a shorter survival time compared with the group with low expression (Wen et al. 2020). In principle, LYTAC technology achieves targeted ABCG2 degradation by recruiting lysosomal degradation pathways, resulting in approximately 63% degradation efficiency *in vitro* (Lu et al. 2023).

Studies using flow cytometry and immunofluorescence under nonpermeabilized conditions have consistently demonstrated that EGFR is predominantly localized on the tumor cell surface, confirming its functional role as a membrane receptor (Tanaka et al. 2018). In parallel, PD-L1 is also expressed on the plasma membrane and can be dynamically regulated by upstream signaling. Notably, activation of EGFR signaling has been shown to significantly upregulate PD-L1 expression, whereas pharmacological inhibition of EGFR leads to a marked reduction in PD-L1 levels (Concha-Benavente et al. 2016). Mechanistically, this regulation is mediated through canonical downstream pathways, such as ERK and AKT, linking oncogenic signaling with immune checkpoint control (Ng et al. 2018). This EGFR and PD-L1 axis establishes a coordinated network that supports tumor cell proliferation while simultaneously promoting immune evasion, thereby contributing to resistance against both tyrosine kinase inhibitors (TKIs) and PD-1/PD-L1 blockade therapies.

Given that both EGFR-driven oncogenic signaling and PD-L1-mediated immune suppression depend on their membrane localization, they represent rational targets for extracellular protein degradation strategies. LYTAC technology leverages lysosome-targeting receptors to selectively internalize and degrade membrane proteins, offering a distinct approach compared to conventional inhibition. For instance, GALNAc or peptide ligand-based LYTACs have been shown to efficiently degrade EGFR, including multiple drug-resistant mutants, as well as other receptors, such as HER2, resulting in suppression of downstream signaling and reversal of TKI resistance (Ahn et al. 2021; Li et al. 2025c). Similarly, LYTAC-mediated degradation of PD-L1 significantly reduces its surface expression and enhances T cell-mediated cytotoxicity (Liu et al. 2024). Importantly, a pooled analysis of over 3800 patients with NSCLC indicates that tumors with higher PD-L1 expression derive greater benefit from PD-1/PD-L1 inhibitors, underscoring the functional relevance of PD-L1 abundance (Vallejo et al. 2024). Collectively, these findings support a coherent therapeutic rationale in which LYTAC degradation of EGFR and PD-L1 may simultaneously disrupt tumor-intrinsic survival pathways and relieve immune suppression, thereby improving overall treatment efficacy.

### 2.3. Intracellular proteins

Intracellular protein networks constitute a central layer of tumor drug resistance, particularly through the regulation of DNA damage repair and apoptotic signaling. One of the mechanisms involves enhanced DNA double-strand break (DSB) repair capacity, which directly attenuates the cytotoxic effects of chemotherapy and radiotherapy. Ubiquitin-dependent regulatory systems, including deubiquitinating enzymes (DUBs) and E3 ligases, play critical roles in stabilizing key repair factors and promoting homologous recombination (HR) efficiency (Dasharathy et al. 2025). For instance, USP7-mediated deubiquitination stabilizes RNF169 at DNA damage sites, thereby facilitating HR repair and contributing to resistance to PARP inhibitors in breast cancer (Soberanis Pina and Lheureux 2023). In parallel, dysregulation of intracellular apoptotic pathways further reinforces resistance phenotypes. Alterations in regulatory proteins, such as JAB1 and COMP, suppress caspase activation and sustain anti-apoptotic signaling, ultimately enabling tumor cells to evade drug-induced cell death (Guo et al. 2023; Papadakos et al. 2021; Wang et al. 2019b). These observations collectively indicate that intracellular resistance is largely driven by protein stability control and signaling network rewiring, highlighting intracellular proteins as critical therapeutic targets.

Indeed, PROTAC technology has demonstrated clear advantages in eliminating such intracellular drivers, particularly those involved in transcriptional regulation, DNA repair, and oncogenic signaling, thereby overcoming resistance mechanisms that are refractory to conventional inhibitors (Burke et al. 2022). A systematic comparison of the pros and cons of PROTACs, LYTACs, and other platforms, focusing on their target space, delivery routes, and degradation efficiency (as summarized in Table 2). However, this approach is inherently restricted to intracellular, soluble, and druggable proteins, leaving extracellular ligands, secreted factors, and membrane-associated resistance mediators inaccessible. Therefore, LYTACs may provide a more comprehensive strategy to dismantle resistance networks across different cellular compartments. In this context, the development of diversified protein degradation technologies represents a critical step toward overcoming the compartmental heterogeneity of tumor drug resistance.

**Table 2.** Degradation platform comparison.

Platform	Target space	Delivery route	Degradation efficiency	Advantages	Limitations	References
PRPTAC	Intracellular proteins and transmembrane proteins with accessible intracellular domains	Passive diffusion	DC_50_ < 10 nM, D_max_ > 85%	Targets ‘undruggable’ proteins via degradation, not inhibition Sub-stoichiometric dosing due to turnover Linker enables fine-tuning of degradation profiles	High molecular weight and polarity can limit cell permeability and oral bioavailability Limited to known E3 ligases Potential off-target effects	Yang et al. (2025)
LYTAC	Extracellular, membrane and intracellular proteins	Receptor mediated endocytosis	Degradation about 50%–90%	Expanded extracellular target scope Modular receptor specific targeting Tissue selective degradation potential	Limited tissue penetration Receptor expression dependency Synthetic complexity	Xiao et al. (2025a, 2025c)
AbTAC	Extracellular surface proteins	Antibody-driven co-engagement of membrane target and transmembrane E3 ligase	DC_50_: 3.4 nM, D_max_: 63%	Dual target E3 engagement High specificity and affinity Extended serum half-life and stability	Limited tissue penetration Complex engineering and high cost Immunogenicity risk	Cotton et al. (2021)
MoDE-A	Extracellular proteins	Hepatocyte targeting via ASGPR	EC₅₀: 7.4 nM and 0.12 μM	Small molecule, non-protein nature Hepatocyte-specific high-capacity degradation Potential immune tolerance	Receptor dependence Limited target scope Route of administration constraints	Fuchs et al. (2024)
Nano LYTAC	Extracellular and membrane Proteins	Receptor-mediated endocytosis of nanoparticle–ligand conjugates	The degradation persistence will be prolonged (50%–70%)	Expanding the target scope Improving pharmacokinetics Multifunctional integration and synergy	Strong dependence on delivery efficiency High synthesis and translational complexity Dependence on external activation	Lin et al. (2025)

## 3. Development of protein-targeted degradation technology

### 3.1. The development of PROTACs

PROTACs are a novel targeting strategy based on inducing target protein degradation. By bridging the target protein with E3 ubiquitin ligase, the target protein is ubiquitinated and then degraded by the proteasome (Burke et al. 2022). Unlike traditional inhibitors, PROTACs have shifted from the traditional inhibition of protein functions to the elimination of protein entities, and they have unique advantages in conquering undruggable targets and solving problems, such as drug resistance. In 2019, ARV-110 (Bavdegalutamide) became the first PROTAC drug to enter clinical trials, designed to selectively target the androgen receptor (AR). In a Phase I study involving patients with metastatic castration-resistant prostate cancer, ARV-110 exhibited a favorable tolerability profile and preliminary evidence of antitumor activity. Furthermore, in the Phase III clinical trials, the oral PROTAC estrogen receptor degrader vepdegestrant (ARV-471), administered as monotherapy, significantly reduced the risk of disease progression or death by 42% compared with fulvestrant in previously treated patients with *ESR1*-mutated, advanced breast cancer (Campone et al. 2025). This platform demonstrated significant advantages in addressing drug resistance. CFT8919 is a PROTAC molecule designed to selectively target and degrade oncogenic EGFR proteins harboring the L858R mutation. A Phase I clinical trial for this compound was initiated in 2023. Compared to traditional EGFR inhibitors, CFT8919 represents a mechanistic advancement by directly degrading mutant EGFR proteins (Zhou et al. 2025). The key features of clinical translation for representative platforms, including PROTACs and LYTACs (as summarized in Table 3). Notably, it demonstrates substantial potential in overcoming resistance mutations that limit the efficacy of second- and third-generation EGFR tyrosine kinase inhibitors.

**Table 3.** Translational maturity and feasibility of different platforms.

Platform	Translational maturity and feasibility	References
PROTAC	Multiple candidates have entered clinical trials, with ARV-471 (ER degrader), vepdegestrant and BGB-16673 (BTK degrader) progressing to Phase III. Clinical safety and preliminary efficacy have been demonstrated across oncology and autoimmune indications, indicating high translational maturity.	Campone et al. (2025); Hamilton et al. (2024); Ren et al. (2025)
LYTAC	No human clinical trials initiated to date. Translational feasibility supported by *in vivo* proof-of-concept studies, but clinical maturity remains low.	Xiao et al. (2025b)
AbTAC	Currently limited to *in vitro* and animal proof-of-concept studies. No registered human clinical trials. Translational feasibility remains exploratory and unvalidated clinically.	Cotton et al. (2021)
MoDE-A	Early clinical translation achieved. Candidates such as BHV-1300 and BHV-1400 have entered Phase I trials, demonstrating target protein reduction (e.g. IgG, Gd-IgA1) in humans. Platform shows emerging clinical feasibility but remains in early-stage development.	Lee et al. (2024)
Nano LYTAC	Restricted to preclinical animal studies. No human clinical trials reported. Translational pathway and scalability remain to be established.	Xu et al. (2024)

### 3.2. The limitations of PROTACs

Despite providing a novel strategy for degrading undruggable targets, PROTACs exhibit several intrinsic limitations. Because they rely on the ubiquitin proteasome system, PROTACs can act only on intracellular soluble proteins (Paudel et al. 2023). For example, although the small molecule PROTAC C8 developed by Yao et al. (2024), selectively degrades intracellular hyperphosphorylated tau and improves cognitive performance in Alzheimer’s disease model mice, extracellular amyloid-β (Aβ) plaques remain inaccessible to this mechanism, highlighting the restricted target scope of PROTACs. Their huge structure, with two ligands connected by linker, poses additional design challenges, as the linker must simultaneously provide sufficient conformational flexibility for ternary complex formation and support adequate cell permeability and pharmacokinetic behavior. Lu et al. (2025a) found that among XL5 based derivatives, only the long-chain-(CH₂)₅-containing XL5-VHL-7 achieved a twofold increase in degradation efficiency, emphasizing the high structural sensitivity of PROTAC activity. Moreover, the typically high molecular weight, substantial polarity, and large hydrophobic surface area of PROTACs lead to poor metabolic stability, limited permeability, and unfavorable pharmacokinetic profiles, as illustrated by the prototype PROTAC 6e reported by Chen et al. (2023), which, despite potent *in vitro* activity against wild-type and mutant BTK, displayed extreme metabolic instability and an impractically short *in vivo* half-life until its linker was replaced with a more rigid scaffold. Collectively, these challenges indicate that targeted protein degradation technologies must continue to diversify degradation mechanisms to broaden their range of addressable targets.

### 3.3. Development of LYTACs

In 2020, Bertozzi et al. systematically proposed the concept of LYTACs, marking a critical expansion of targeted protein degradation strategies from intracellular proteins to extracellular and membrane associated proteins (Banik et al. 2020). In their study, LYTAC molecules were generated by coupling target binding ligands with mannose-6-phosphate (M6P) oligomers to specifically activate the CI-M6PR-mediated endocytic pathway, thereby directing secreted or membrane proteins to lysosomes for degradation. CI-M6PR is considered a versatile degradation platform due to its broad tissue expression, but it also lacks targeting specificity. The recycling of surface receptors​ is its rate limiting step. In the hepatocellular carcinoma cell line HepG2, the degradation efficiency​ of CI-M6PR based LYTACs against the IgG model remains limited. To mitigate potential off-target toxicity associated with the broad expression of CI-M6PR in normal tissues while improving the tumor targeting specificity of the therapeutic strategy, Ahn et al. (2021) reported the GalNAc-LYTAC platform, in which trimerized N-acetylgalactosamine (GalNAc) ligands were conjugated to target antibodies to exploit the ASGPR. The specific expression of ASGPR on hepatocytes is expected to allow for the targeted degradation of membrane proteins on liver cells. Compared with CI-M6PR-mediated LYTACs, GalNAc-based ASGPR-LYTACs exhibit significantly higher internalization efficiency in the same HEPG2 cell model. However, due to the restricted expression of ASGPR in hepatocytes, the application of this strategy is largely limited to liver-associated diseases.

In one study, Yoda's group developed a bispecific DNA aptamer-based HER2-LYTAC capable of simultaneously binding HER2 and IGF2R (CI-M6PR), thereby inducing lysosomal degradation of HER2 and effectively suppressing cancer cell proliferation (Yoda et al. 2025a). Another study coupled gefitinib to IGF2R aptamers to construct an EGFR-LYTAC capable of simultaneously degrading wild type and multiple mutant forms of EGFR, and demonstrated superior antitumor efficacy compared to osimertinib in both *in vitro* and *in vivo* models (Li et al. 2025b). Collectively, the evolution of LYTACs from early M6P-CI-M6PR designs to GalNAc-mediated tissue targeting and alternative scaffolds has established LYTACs as a modular and versatile platform for degrading extracellular and membrane proteins.

### 3.4. Optimization of linkers

In LYTACs, the linker is a critical structural element connecting the target-binding and lysosome-targeting ligands. Its length, flexibility, and physicochemical properties directly impact target engagement, receptor recruitment, cellular permeability, and stability, thereby playing a decisive role in degradation efficiency. To address acquired resistance arising from EGFR mutations during treatment with EGFR tyrosine kinase inhibitors, Li et al. designed EGFR-targeting LYTAC molecules and investigated the interaction between erlotinib–linker analogs and EGFR using molecular dynamics simulations. The results indicated that introduction of the linker altered the binding conformation of erlotinib, causing its key functional groups to rotate outward. However, stable binding was still maintained in the exon 19 deletion mutant EGFR system. Degradation assays further demonstrated that a linker containing five thymine units exhibited the highest degradation efficiency. Notably, the molecule LY-dE#5 achieved up to about 80% EGFR degradation in A549 cells at 2 μM and significantly inhibited tumor growth in BALB/c nude mouse xenograft models (Li et al. 2025c). Nevertheless, because the IGF2R is widely expressed in normal tissues, potential on-target toxicity remains a concern. A similar structural activity relationship has been observed in HER2-LYTAC systems. Extending the linker length (approximately 5 bp longer than the original construct) enhanced HER2 degradation activity while maintaining comparable binding affinity (Yoda et al. 2025b). However, simply increasing the length of nucleic acid linkers may introduce excessive molecular rigidity and limit tissue permeability. In addition, LYTAC molecules exhibit limited stability in serum-containing media, with a half-life of only about 6 h, highlighting the necessity for further linker optimization.

Extensive studies in the targeted protein degradation field, particularly in PROTAC systems, provide valuable insights for rational linker design in LYTACs. For example, molecular dynamics simulations and structural modeling predicted that a pentyl linker [–(CH₂)₅–] facilitates formation of a stable ternary complex, experimental validation further showed that the derivative XL5-VHL-7 exhibited approximately twofold greater apoptosis-inducing activity than the parent PROTAC molecule (Lu et al. 2025b). Moreover, in VHL-recruiting PROTACs, replacing two methylene units in an alkyl linker with oxygen atoms to generate a polyethylene glycol (PEG) linker allows the molecule to maintain a folded conformation in the nonpolar membrane environment similar to that in polar media, thereby preserving high membrane permeability (Poongavanam et al. 2025). Collectively, insights from PROTAC linker engineering provide a rational framework for LYTAC optimization. Integrating AI-driven molecular design with PEG-based linker engineering may enable systematic optimization of linker length, flexibility, and physicochemical properties, ultimately improving degradation efficiency and *in vivo* stability of next-generation LYTAC molecules.

### 3.5. Advantages of LYTACs over PROTACs in reversing tumor drug resistance

Compared with PROTAC technology, which depends on engagement of the UPS, LYTACs exploit receptor-mediated endocytosis and lysosomal degradation pathways to enable the direct elimination of extracellular and membrane proteins. By mediating the complete degradation of the entire target protein, LYTACs can overcome drug resistance caused by persistent receptor tyrosine kinase activation, bypass receptor mediated compensatory signaling, or resistance-associated mutations that are inaccessible to PROTACs. Structurally, LYTACs adopt a modular bifunctional architecture composed of a POI moiety and a LTR ligand, allowing flexible targeting of diverse membrane and secreted proteins without the need for intracellular delivery or cell permeability optimization. Functionally, by removing the resistance driver at its extracellular or membrane proteins, LYTACs not only suppress primary oncogenic signaling but also concurrently attenuate parallel and compensatory signaling networks, thereby reducing pathway redundancy and delaying adaptive resistance. In addition, the degradation of surface immune checkpoints or immunosuppressive ligands may partially alleviate tumor immune evasion, supporting potential synergy with immunotherapies (Liu et al. 2024).

In LYTAC technology, the key lysosome-targeting receptor can be recycled from the endo-lysosomal system and re-trafficked to the plasma membrane following target protein internalization and delivery, enabling multiple rounds of target degradation. Using a genome-wide CRISPR knockout screening, the researchers identified the retromer complex as a key regulator of LYTAC mediated degradation. Mechanistically, the retromer complex mediates CI-M6PR recycling, thereby trafficking the LYTAC target receptor complex from endosomes back to the plasma membrane and limiting its transport to lysosomes. Functional validation was performed by genetic ablation of a core retromer subunit (e.g. VPS26A) in an EGFR-LYTAC degradation system. Western blot analysis showed that the degradation efficiency of the target protein increased from approximately 70%–80% in wild-type cells to more than 90% in retromer-deficient cells (Ahn et al. 2023). These findings indicate that receptor recycling constitutes a critical trafficking checkpoint that constrains LYTAC efficacy and suggest that modulation of retromer-dependent pathways may represent a potential strategy for improving LYTAC-mediated protein degradation. Overall, LYTACs provide a complementary and more effective alternative to PROTACs for addressing tumor drug resistance.

## 4. Lysosome-directed TPD technologies: mechanisms and design principles

### 4.1. MoDE-As

MoDE-A is a small-molecule LYTAC platform that mediates lysosomal degradation of extracellular proteins through ASGPR targeting. In the design by Caianiello and colleagues, a carboxylate-terminated macrophage migration inhibitory factor (MIF) tautomerase inhibitor is linked via a PEG linker to a tri‑valent GalNAc moiety (Caianiello et al. 2021). D‑MoDE‑A shows high binding and delivery efficiency, with half‑maximal association with the ASGPR receptor occurring at concentrations of 7.4 nM and 0.12 μM. Its small‑molecule nature may also support favorable tissue penetration. Importantly, repeated administration of D‑MoDE‑A over 21 days did not induce detectable anti‑drug antibodies, indicating that such small molecules are less immunogenic than peptides and may reduce the risk of anti‑drug antibody‑associated resistance (Fuchs et al. 2024). This platform leverages the high, constitutive expression of the ASGPR on hepatocytes, making it particularly effective for liver-directed applications, such as hepatocyte-specific drug delivery and clearance of circulating proteins. However, its applicability is constrained by this very specificity. The predominant localization of ASGPR to hepatocyte surfaces limits its utility in most solid tumors, which largely lack this receptor. Furthermore, its short serum half-life (0.67 h) restricts sustained systemic exposure and broader tissue distribution. Therefore, future optimization efforts should focus on extending its *in vivo* half-life, broadening the receptor targeting range, and enhancing its utility across diverse tissues.

### 4.2. AbTACs

A major resistance mechanism in immune checkpoint therapy arises from persistent or re-expressed PD-L1 on tumor cells, since conventional antibodies only block PD-1/PD-L1 interaction without eliminating the protein. Antibody-based modular technologies, including antibody–drug conjugates (ADCs), antibody-targeted chimeras (AbTACs), and molecular glue–mediated systems, have emerged as powerful platforms for targeted therapeutic intervention. Despite sharing a common foundation in antibody-guided specificity, these modalities exhibit fundamentally distinct mechanistic principles, particularly in their modes of target engagement, effector recruitment, and downstream biological outcomes (as summarized in Table 4). To generate AbTACs, an Fc-engineering strategy was employed to construct a bispecific IgG. Specifically, the knobs-into-holes approach was used to promote selective heterodimerization of two distinct heavy chains, thereby minimizing heavy-chain mispairing. To avoid light-chain mispairing, the heavy chain–light chain pairs targeting RNF43 and PD-L1 were expressed separately as half-IgGs and subsequently assembled *in vitro* to produce the bispecific antibody AC-1. A His-tag was introduced on the knob half-antibody to enable affinity purification and remove hole-hole homodimer byproducts, improving product purity. Functionally, AC-1 induced efficient degradation of cell-surface PD-L1 in multiple cancer cell lines, including MDA-MB-231, HCC827, and T24, demonstrating robustness across different cellular contexts. In MDA-MB-231 cells, AC-1 showed potent activity with a degradation concentration 50% of 3.4 nM and a maximum degradation of 63%. Although the IgG format may confer pharmacokinetic advantages due to FcRn-mediated recycling, no *in vivo* pharmacokinetic studies were performed, and its pharmacokinetic properties remain to be determined (Cotton et al. 2021). By recruiting membrane E3 ligases (e.g. RNF43 or ZNRF3) via bispecific antibodies, AbTACs enable targeted degradation of cell-surface proteins, thereby overcoming the intracellular target restriction of traditional PROTACs while offering opportunities to reduce resistance and broaden target scope through E3 ligase diversity, epitope exchange, and modular antibody engineering.

**Table 4.** Comparison of targeting modalities.

Modality	Construction	Target space	Targeted binding	Cellular pathway	Mechanism of action
ADC	Monoclonal antibody Linker Cytotoxic payload	Cell surface antigens	Antigen-antibody binding	Endocytosis lysosomal pathway	Delivers a cytotoxic drug directly into the target cell and induce cell death
AbTAC	A fully recombinant bispecific antibody with two distinct antigen-binding sites	Membrane proteins	Induced proximity to membrane-bound E3 ligases	Endocytosis lysosomal pathway	Degrades specific cell surface proteins
Molecular glue	A single, monovalent small molecule that typically contains integrated pharmacophores	Intracellular proteins	Small molecule induced protein–protein interaction	Ubiquitin–proteasome system	Promotes ubiquitination and proteasomal degradation of the target protein
LYTAC	Target protein ligand Lysosome transport receptor ligand Linker	Extracellular and membrane proteins	Lysosome targeting receptor engagement	Endocytosis lysosomal pathway	Redirects extracellular and membrane proteins to the lysosome for degradation

### 4.3. Nanosized LYTACs

Nanosized LYTAC systems (nano-LYTACs) integrate nanotechnology with targeted protein degradation to enhance drug delivery and therapeutic efficacy in tumors (Kunachowicz et al. 2025).

Xu et al. (2024) developed a semiconducting polymer nano-LYTAC functionalized with IL-4 receptor (IL-4R) targeting peptides and lysosome-sorting peptides on a single nanoparticle backbone. SPNly nanoparticles were engineered for prolonged tumor retention, demonstrating high intratumoral localization for over 48 h. This directly translated into favorable pharmacokinetics, as evidenced by *in vivo* imaging showing efficient tumor accumulation and a sustained high tumor to liver ratio for up to 72 h. This might be related to the enhanced permeability and retention effect of the nanocarriers. Importantly, under ultrasound activation, these SPNly nanoparticles generate cytotoxic ROS to eliminate cancer cells while simultaneously targeting the immunosuppressive IL-4R on M2 macrophages for lysosomal degradation. In another study, Wang et al. designed a GalNAc-modified nanosphere LYTAC loaded with an anti-CD24 antibody and the enzyme glucose oxidase. Glucose oxidase can consume glucose within the tumor and generate additional oxidative stress. The two work in synergy, which is expected to enhance the killing effect on cancer cells (Wang et al. 2023). The synergistic strategy of Nanosphere LYTAC resulted in the degradation of the target protein approaching 85%–90% within 48 h *in vitro* experiments. However, the synthesis of nanoparticles remains complex, and maintaining monodispersity during scale-up production poses significant difficulties. Coupled with inter-patient metabolic variations and tumor microenvironment heterogeneity, these factors hinder the establishment of universal dosing regimens, presenting ongoing challenges for clinical translation. The enhanced permeability and retention (EPR) effect exhibits substantial heterogeneity and is often weaker in human tumors than in preclinical models. Emerging strategies, such as active targeting ligand modification or the development of self-propelled nanorobots capable of autonomous navigation toward tumor tissues, may help enhance tumor accumulation and mitigate the limitations of EPR-dependent delivery (Fan et al. 2023). The clinical translation of these different strategies has progressed heterogeneously (as summarized in Table 3).

## 5. Applications of LYTACs in overcoming cancer drug resistance

### 5.1. Immune system regulation

#### 5.1.1. Targeted degradation of PD-L1

​Antibody blockers inhibit the interaction between PD-L1 and PD-1 ligands by binding to them. However, when dealing with tumor heterogeneity, dynamic changes in expression levels, and regulation by the tumor microenvironment, primary or acquired resistance often occurs (Liu et al. 2024). In one study, Xiao et al. (2025a) proposed folate receptor*-α* (FRα) -targeting chimeras (FRTACs), which were constructed by conjugating multivalent folate molecules with targeting antibodies via a PEG linker to form FR-Atz. Degradation strategies eliminate the target protein rather than transiently blocking ligand receptor interactions, thereby more effectively suppressing signaling. Western blotting confirmed that FR-Atz achieved over 80% degradation of PD-L1 at a degradation concentration 50% of 0.080 nM. Target degradation may produce longer-lasting pharmacodynamic effects compared with transient antibody blockade. Immunofluorescence imaging further showed that 48 h of FR-Atz treatment nearly eliminated surface PD-L1, outperforming PBS and antibody-only controls. *In vivo*, Dylight 680-labeled FR-Atz accumulated efficiently in H292 tumors after intravenous administration and markedly inhibited tumor growth. The anti-tumor efficacy was next evaluated in an H292 human lung cancer xenograft mouse model. Immunohistochemical analysis verified that FR-Atz exerts a powerful anti-tumor effect by efficiently degrading the PD-L1 protein in tumor tissues and inducing tumor cell apoptosis. By reducing the need for sustained receptor occupancy, degradation strategies may allow lower dosing and potentially mitigate systemic toxicity. Moreover, tumor microenvironment analyses in xenograft tissues revealed a shift in tumor-associated macrophages from an M2 to M1 phenotype, suggesting that PD-L1 degradation reshapes local immune polarization. A notable consideration for LYTAC efficacy is its dependence on the engagement of both FR and PD-L1 receptors, which introduces a potential challenge due to the possible heterogeneity in the expression levels of these endocytic receptors.

In another study, covalent Pep-TACs, leveraging stable covalent interactions, exhibit stronger PD-L1 binding affinity, higher stability, and superior degradation efficiency compared to their non-covalent counterparts (Xiao et al. 2025b). This property enables them to cross the blood–brain barrier via receptor-mediated transport, demonstrating promising therapeutic potential in brain tumor models. However, biodistribution studies following intraperitoneal injection reveal their widespread accumulation in multiple tissues, including the brain, liver, and kidneys. This broad tissue distribution raises concerns regarding potential off-target toxicity. Sustained accumulation in normal tissues may lead to adverse effects, representing a key safety risk that requires careful evaluation during clinical translation.

#### 5.1.2. Targeted degradation of CD24

**Figure 2.**
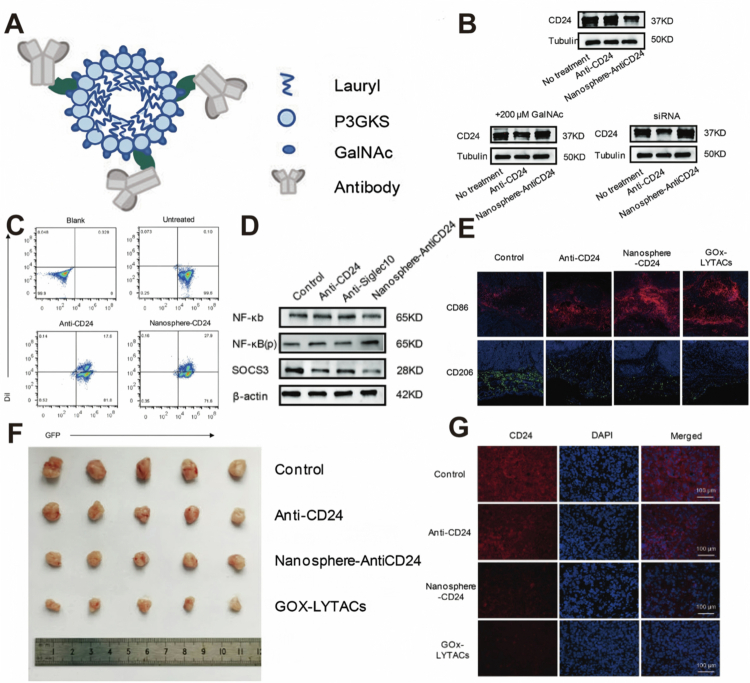
(A) Structure of Nano-LYTACs. (B) Western blot confirmed that Nano-LYTACs degrade CD24. (C) Fluorescence microscopy demonstrated that NanosphereAntiCD24 potently enhances the phagocytosis of red-labeled HepG2 cells by green-labeled THP-1 (GFP⁺) macrophages. (D) Siglec-10 downstream NF-κB, *p*-NF-κB, and SOCS3 levels after treatment with Anti-CD24. (E) Immunofluorescence images revealed a marked increase in the M1 marker CD86 (red) and a concomitant decrease in the M2 marker CD206 (green) within tumors, correlating with enhanced phagocytic potential. (F) Picture of a dissected hepatoma tumor from mice 28 days after inoculation. (G) Immunofluorescence images proved that the intensity of red fluorescence in the GOx-LYTACs group was lower. Reprinted with permission from Wang et al. (2023). Copyright 2023, Wiley-VCH GmbH.

Traditional antibody blockers prevent the interaction between CD24 and Siglec-10, but the CD24 is still present on the surface of tumor cells, which may restore the inhibitory signal for macrophage phagocytosis due to a decrease in antibody concentration or insufficient receptor occupancy (Barkal et al. 2019). To address this, Wang et al. developed nanosphere-antiCD24 LYTACs, in which GalNAc-modified peptide nanospheres were conjugated to anti-CD24 antibodies to induce ASGPR-mediated internalization and lysosomal degradation (Figure 2A) (Wang et al. 2023). In HepG2 cells, treatment with 100 nM nanosphere-antiCD24 for 24 h achieved approximately 60%–70% degradation of membrane CD24 (Figure 2B). Functionally, co-culture of treated HepG2 cells with GFP⁺ M1-like THP-1 macrophages resulted in a marked increase in double-positive cell populations, indicating enhanced macrophage-mediated phagocytosis (Figure 2C). Mechanistically, analysis of downstream components of the CD24/Siglec-10 pathway revealed increased phosphorylation of NF-κB alongside decreased SOCS3 expression, while total NF-κB levels remained unchanged (Figure 2D). Importantly, by eliminating CD24 protein from the tumor cell surface instead of transiently blocking its interaction with Siglec-10, this degradation-based strategy reduces the likelihood of signal restoration due to decreased antibody occupancy.

To further enhance therapeutic efficacy, glucose oxidase (GOx) was encapsulated within the nanospheres to generate GOx-LYTACs, thereby integrating targeted CD24 degradation with glucose deprivation-based starvation therapy. In tumor-bearing mice, immunofluorescence analysis of tumor tissues showed that GOx-LYTACs reduced CD24 expression *in vivo* (Figure 2E). Moreover, macrophage polarization shifted toward an M1 phenotype, as evidenced by increased CD86 and decreased CD206 staining. In a HepG2 xenograft model, the GOx-LYTACs group exhibited the greatest suppression of tumor growth over a 21-day observation period compared with control and single-treatment groups (Figure 2F, G). However, evidence is based primarily on xenograft models in immunodeficient mice rather than immunocompetent hepatocellular carcinoma models, limiting conclusions regarding durable antitumor immunity.

### 5.2. Targeted degradation of EGFR

The on-target resistance mechanisms to the third-generation EGFR-TKI osimertinib primarily involve two pathways. First, the acquired C797S mutation in EGFR, which abrogates the covalent binding essential for drug action. Second, EGFR gene amplification in tumor cells, which leads to receptor overexpression and confers resistance through a gene dosage effect that partially overcomes TKI suppression (Leonetti et al. 2019). To counteract this, Pan et al. (2025) developed a lysosome-targeting chimera (s-LYTAC) by fusing an engineered IGF2 mutant (IGF2-M5.6) to a cetuximab heavy chain, generating Cet-sLYTAC. Compared to traditional wild-type IGF2, which may simultaneously bind to IGFF1R and activate its tyrosine kinase signaling pathway with potential tumorigenic risks, this technology employs engineered IGF2-M5.6 to demonstrate high-affinity binding to IGF2R. In TKI-resistant NCI-H1975 cells, treatment with 50 nM Cet-sLYTAC achieved over 90% degradation of EGFR. In a JIMT-1 xenograft model, intraperitoneal administration of Ptz-sLYTAC resulted in a reduction of HER2 and EGFR levels, effectively overcoming trastuzumab resistance associated with MUC4 overexpression. IGF2R (M6PR) is a multifunctional transmembrane receptor. Beyond mere ligand binding, it critically regulates lysosomal trafficking, growth factor activity, and cell growth suppression. This functional pleiotropy implies that its chronic or high-level modulation may carry unforeseen risks of systemic side effects.

**Figure 3.**
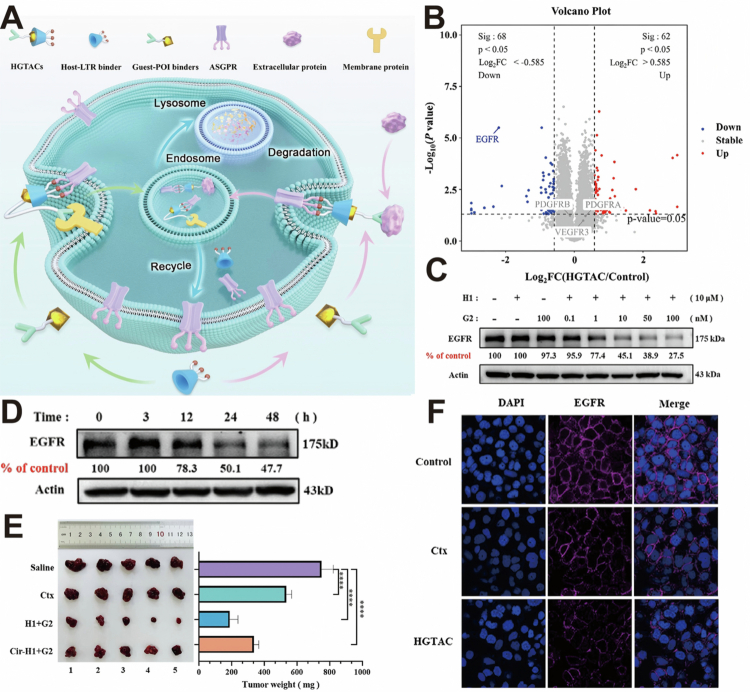
(A) Mechanism of HGTACs. (B) The volcano plot displayed the proteomic profile of quantified proteins in HepG2 cells following HGTACs treatment, from which EGFR emerged as one of the most significantly downregulated proteins. (C) Western blotting showed the degradation of EGFR by HGTAC. (D) HGTAC time-dependently degraded EGFR in Western blotting. (E) The picture showed that the HGTACs (G2 ± H1) led to a marked reduction in tumor burden. (F) Confocal microscopy confirmed EGFR degradation. Reprinted with permission from Chen et al. (2025). Copyright 2025, Wiley-VCH GmbH.

Beyond ligand engineering, linker optimization represents another critical strategy for improving degradation efficiency. Chen et al. (2025c) proposed host-guest bridged LYTACs (HGTACs). In this design, β-cyclodextrin (β-CD) is attached to the degradation receptor ligand to form the host module, and Adamantane is attached to the target protein ligand (Cetuximab) to form the guest module. The two are non-covalently linked through the β-CD-ADA host–guest interaction to form HGTACs (Figure 3A). This design mitigates single-point mutation-induced complete drug resistance and enables flexible ligand stoichiometric adjustment, evading the dose-dependent hook effect. The research found that when the ratio of H1 to G1 is approximately 5:1, the highest endocytosis efficiency can be achieved. Quantitative proteomic analysis of HepG2 cells treated with HGTACs for 48 h showed a marked degradation of EGFR levels, while VEGFR, PDGFRα, and PDGFRβ expression remained largely unchanged (Figure 3B). In HepG2 cells, HGTACs achieved over 50% EGFR degradation at 10 nM, reaching maximal effects at 100 nM without showing a pronounced ‘hook effect’ (Figure 3C). HGTAC-induced EGFR degradation is concentration and time dependent (Figure 3D). In a HepG2 xenograft mouse model, HGTAC treatment significantly inhibited tumor growth by day 21, as indicated by reduced tumor volume and weight compared to control animals (Figure 3E). Co-localization immunofluorescence analysis of HepG2 cells confirmed that the membrane EGFR signal was significantly reduced after HGTACs treatment and was superior to the effect of Ctx alone (Figure 3F). This paradigm shift from protein function inhibition to complete protein depletion circumvents the impact of kinase domain mutations in its mechanism of action and directly addresses the issue of protein overexpression.

This linker design achieves precise regulation of degradation efficiency through stoichiometric ratio adjustment and competitive ligand intervention. The core design of HGTACs relies on the ASGPR as the LTR. This receptor is highly expressed on hepatocytes, which limits the general applicability of HGTACs. Furthermore, the chemical conjugation of antibodies with synthetic linkers for the POI ligand moiety may pose a risk of immunogenicity. Addressing these limitations will be an important focus of future formulation and pharmaceutical development studies.

### 5.3. Targeted degradation of CTGF

**Figure 4.**
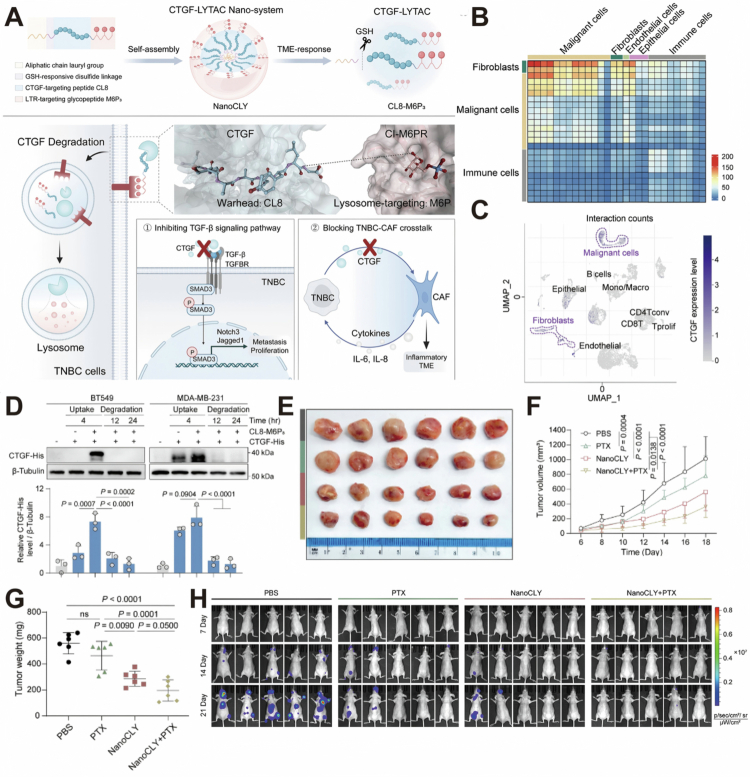
(A) Nano-LYTAC mediated the degradation of CTGF. (B) Cell–cell interactions between different cell clusters within the TNBC TME. (C) UMAP plots demonstrated that both TNBC cells and CAFs highly expressed CTGF. (D) Western blotting showed the degradation of CTGF by nano-LYTAC. (E) Photographs of tumors excised post-treatment. (F) Tumor growth was measured during the treatment period. (G) Tumor weights were measured after treatment. (H) *In vivo* imaging of mice showed that NanoCLY + PTX inhibits bone metastasis. Reprinted with permission from Lin et al. (2025). Copyright 2025, Wiley-VCH GmbH.

Previous findings indicated that connective tissue growth factor (CTGF) signaling upregulates anti-apoptotic proteins, such as BCL-xL, thereby reducing chemotherapy-induced cell death (Wang et al. 2019a). By boosting glycolysis through Glut3, CTGF alters tumor metabolism in a way that allows cancer cells to better withstand treatment-induced stress, which in turn facilitates the development of resistance to chemotherapy and targeted drugs (Kim et al. 2021). In a study, Lin et al. (2025) developed CTGF-LYTAC for targeted degradation of extracellular CTGF. This strategy degrades CTGF in an LYTAC-dependent manner, blocks CTGF-Glut3-driven glycolytic metabolic reprogramming, and thereby attenuates the adaptive capacity of tumor cells to therapeutic stress, which may enhance their chemotherapeutic sensitivity. This platform was constructed by conjugating an octapeptide ligand targeting CTGF (CL8) with a high-affinity glycopeptide ligand containing M6P₃ via a PEG₃ linker, generating Nano-CLY (CL8-M6P₃) (Figure 4A). Single-cell RNA sequencing of the TNBC tumor microenvironment revealed strong interactions between malignant TNBC cells and cancer-associated fibroblasts (Figure 4B), with both cell types exhibiting high CTGF expression (Figure 4C). Such complex cell–cell interactions are recognized as a central mechanism driving therapeutic resistance in TNBC. In the TNBC cell model, the Western blot results showed that after treatment with Nano-CLY, the CTGF level gradually decreased, indicating that it was degraded by the lysosomal pathway (Figure 4D). Furthermore, the treatment of TNBC with paclitaxel (PTX) is hindered by acquired drug resistance. In the *in vivo* TNBC xenograft model, the combination therapy of Nano CLY degrading CTGF and PTX chemotherapy drugs significantly inhibited tumor growth, and no significant weight loss was caused. Quantitative results showed that the tumor suppression rate exceeded 70% when combined. This indicates antitumor potential in murine models (Figure 4E–G). In the *in vivo* model of bone metastatic breast cancer, the combined treatment group of Nano CLY and PTX was detected by *in vivo* bioluminescence imaging to exhibit the strongest anti-metastasis effect (Figure 4H).

The degradation of CTGF and the downregulation of related signals can reprogram intracellular signaling pathways and promote TME remodeling. This nanotechnology strategy induces lysosomal degradation of CTGF and demonstrates notable antitumor and antimetastatic effects across multiple murine models. However, CTGF is expressed on various cell types and participates in complex interactions within the tumor microenvironment, which may pose potential challenges for therapeutic specificity and safety. In addition, the long-term toxicity profile and potential immunogenicity of this structural platform remain to be systematically evaluated. These factors may therefore represent important considerations in its future clinical translation.

### 5.4. Targeted degradation of c-Met

In therapies targeting the c-Met, drug resistance frequently arises from secondary mutations in the c-Met gene as well as the activation of compensatory bypass signaling pathways. Duan et al. (2024) introduced a chemically engineered platform of multivalent aptamer-based LYTACs (Apt LYTACs). In this design, streptavidin (SA) was used as a scaffold to simultaneously conjugate biotinylated poly-mannose-6-phosphate polymers (bpM6P), targeting LTR, and the Met-specific aptamer SL1. The binding ability of bpM6P-SL1-SA to MDA-MB-231 cells was analyzed and characterized to prove the feasibility of this technology in degrading Met. By adjusting the molar ratio of SA to SL1 (1:3), Apt LYTACs achieved potent Met degradation in MDA-MB-231 cells. Furthermore, multitarget Apt LYTACs were developed to simultaneously degrade PTK7 and Met. The strategy, leveraging its modular, customizable, and multivalent ligand design, provides a proof-of-concept and a practical approach for constructing single-molecule drugs capable of simultaneously degrading multiple synergistic or compensatory targets.

In a recent report, Chen et al. (2025a) engineered a multivalent DNA lysosome-targeting chimera (mSL1-LYTAC) with backbone phosphorothioate (PS) modifications to greatly enhance aptamer stability. When HGF-stimulated HepG2 hepatoma cells were treated with 50 nM mSL1-LYTAC, the phosphorylated forms of c-Met, Akt, and Erk1/2 dropped sharply compared to cells treated with SL1 aptamer or poly-G controls. This striking reduction indicated that the stable mSL1-LYTAC not only binds c-Met with high affinity but also actively shuttles it into lysosomes for degradation, thereby potently silencing the downstream HGF/c-Met signaling cascade. Consistent with this, confocal imaging showed that mSL1-LYTAC strongly inhibited HGF-induced pseudopod extension and actin remodeling, suppressing the enhanced migratory behavior normally driven by c-Met. Importantly, the PS chemistry also conferred prolonged circulation time and tumor targeting capability *in vivo*. In HepG2 xenograft mice, intravenous injection of Cy5-labeled PS-mSL1-LYTAC resulted in the rapid and selective accumulation of the probe at the tumor site, reflecting the enhanced half-life and tumor enrichment afforded by PS modification. Similarly, in an A549 lung cancer xenograft, repeated dosing with mSL1-LYTAC-tPS yielded significantly smaller tumors.

The multivalent DNA LYTAC strategy significantly reduces the drug resistance of the target c-Met gene caused by mutations by packaging and degrading the target. In addition, the scalability and *in vivo* pharmacokinetic behavior of multivalent DNA aptamer systems may require further consideration for clinical translation. The current platform relies on the self-assembly of multiple oligonucleotide components through programmable DNA amplification, which may introduce additional complexity in large-scale manufacturing and require careful optimization to ensure reproducibility and batch consistency. At the same time, the pharmacokinetic properties and *in vivo* durability of DNA aptamer constructs remain to be fully characterized.

### 5.5. Targeted degradation of ABCG2

**Figure 5.**
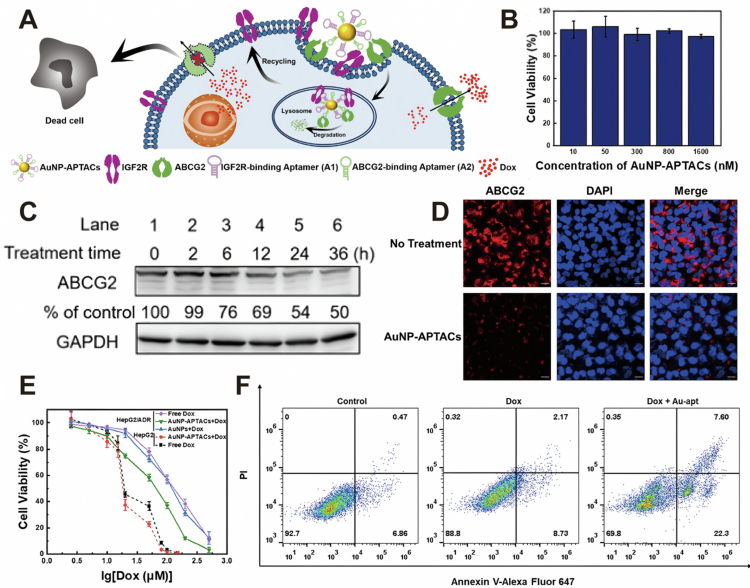
(A) Mechanism of action of AuNP-APTACs. (B) Cell viability of HepG2/ADR cells with AuNP-APTACs treatment. (C) Western blotting assays showed the degradation of ABCG2 by AuNP-APTACs. (D) CLSM showed the degradation of cell surface ABCG2 on HepG2/ADR cells following AuNP-APTACs treatment. (E) IC_50_ assays showed that AuNP-APTACs sensitized cells to Dox. (F) Flow cytometry was used to quantify the percentage of cell death and apoptosis in HepG2/ADR cells treated with Dox in the presence or absence of AuNP-APTACs. Reprinted with permission from Lu et al. (2023). Copyright 2023, Royal Society of Chemistry.

Drug efflux pumps are key contributors to cancer multidrug resistance. Their complex multidomain structures make them difficult to degrade, yet efficient elimination offers great potential for reversing resistance. LYTAC technology provides a powerful means to achieve such targeted protein degradation. In one study, Lu et al. (2023) developed hypervalent bispecific gold nanoparticle-anchored aptamer chimeras (AuNP-APTACs), in which gold nanoparticles serve as a multivalent scaffold functionalized with aptamer chimeras targeting both IGF2R and ABCG2 (Figure 5A). AuNP-APTACs exhibited negligible cytotoxicity toward HepG2/ADR cells (Figure 5B). AuNP-APTACs achieved approximately 63% ABCG2 degradation *in vitro*, as confirmed by immunoblotting (Figure 5C). Confocal microscopy further demonstrated a marked decrease in surface ABCG2 expression following exposure to AuNP-APTACs (Figure 5D). Functionally, AuNP-APTAC-mediated ABCG2 degradation significantly enhanced the intracellular accumulation of doxorubicin (Dox) and restored drug sensitivity. Specifically, the half maximal inhibitory concentration of Dox in HepG2/ADR cells decreased from 136 μM to 65 μM (Figure 5E). In addition, flow cytometric analysis revealed increased apoptosis and necrosis in Dox-treated cells, indicating an improved therapeutic response against drug-resistant hepatocellular carcinoma (Figure 5F).

This strategy addresses the problem of drug resistance caused by insufficient intracellular drug concentration by degrading the drug efflux pump ABCG2. However, its effectiveness has not yet been further verified *in vivo*. The distribution of this strategy within the organism and its potential toxic effects on normal tissues still require systematic and comprehensive assessment through animal experiments and subsequent clinical trials. Moreover, the synthesis process of gold nanoparticles is more complex compared to that of a single small molecule drug, which poses challenges for achieving standardized, low-cost, large-scale production.

### 5.6. Targeted degradation of other drug-resistant proteins

The marked heterogeneity of tumors gives rise to highly diverse pathogenic surface protein repertoires, underscoring the need for LYTAC strategies with exquisite target selectivity. To date, numerous disease-relevant targets and potential indications remain to be systematically explored. Tetrahedral DNA Framework-based LYTACs (TDF-LYTACs) exploit a tetrahedral DNA nanostructure (TDF) as a scaffold, with distinct vertices functionalized by a target-binding aptamer, an IGFIIR-binding aptamer, and an APE1-activated ATP-responsive module (Ning et al. 2025). In MCF-7 cells, TDF-LYTACs achieved approximately 80% CTGF degradation after 8 h of treatment. Fluorescence and Western blot analyses showed that PDGF degradation was enhanced under high ATP conditions and inhibited under low ATP levels, indicating that TDF-LYTAC-mediated degradation is energy-dependent. This ATP-sensitive behavior enables TDF-LYTACs to dynamically modulate degradation efficiency for optimized therapeutic outcomes.

**Figure 6.**
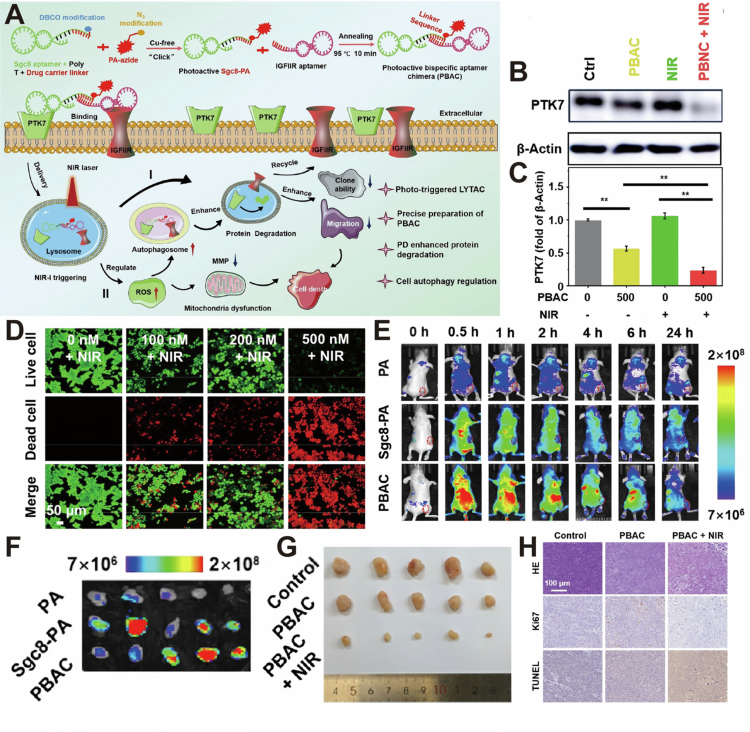
(A) Synthesis and mechanism of PT-LYTAC. (B, C) Western blotting showed that PT-LYTAC degraded PTK7. (D) Fluorescence images of HCT 116 cells treated with PBAC and NIR showed heightened red fluorescence. (E) *In vivo* fluorescence images of signal intensity in HCT 116 tumor-bearing mice at indicated time points. (F) Analysis of NIR fluorescence *in ex vivo* tumors extracted 24 h post-injection. (G) Imaging of tumors harvested from each group on day 11. (H) H&E demonstrated the PBAC + NIR treatment led to massive necrosis, loss of proliferative capacity (Ki67), and widespread apoptosis (TUNEL⁺). Reprinted with permission from Zhang et al. (2025). Copyright 2025, American Chemical Society.

In parallel, Phototriggered LYTACs (PT-LYTACs) introduce a photosensitizer module into a bispecific DNA aptamer chimera (PBAC), with one arm binding IGF2R and the other targeting PTK7 (Figure 6A) (Zhang et al. 2025b). Upon near-infrared (NIR) irradiation, the photosensitizer generates ROS, which synergize with autophagy to enhance lysosomal degradation. Treatment of HCT 116 cells with PBAC + NIR achieved 80% degradation of PTK7 protein, while PBAC alone mediated 40% to 60% degradation, indicating that PD treatment enhances degradation efficiency (Figure 6B, C). *In vitro* heightened red fluorescence showed increased red-fluorescent dead HCT116 cells after PBAC treatment, confirming the effective cell killing activity of this degradation strategy (Figure 6D). In HCT116 tumor-bearing mice, intravenous PBAC injection produced a rapid and strong fluorescence signal at the tumor site by 0.5 h post-asinjection, significantly higher than the signals in mice treated with PA or Sgc8-PA controls (Figure 6E). *Ex vivo* imaging of harvested organs confirmed that PBAC's fluorescence was highest in tumor tissue (Figure 6F), indicating rapid and sustained tumor localization. Therapeutic studies in the HCT116 xenograft model demonstrated that the treatment strategy of PBAC combined with 660 nm near-infrared irradiation showed a significant inhibitory effect on tumor growth during the 11-day observation period, which was significantly superior to other treatment methods (Figure 6G). Additionally, H&E staining confirmed extensive cellular damage and necrosis in tumors from the PBAC + NIR group (Figure 6H). Taken together, these findings demonstrated that the PT-LYTAC approach significantly enhances photodynamic therapy efficacy through targeted protein degradation, resulting in superior antitumor outcomes *in vivo*.

PT-LYTAC combines light responsive design through the lysosome pathway and simultaneously reduces the overall level of target proteins and provides a temporally controllable treatment method. The selective degradation of membrane proteins is expected to overcome the resistance problems caused by target mutations, activation of alternative signals, or overexpression of proteins in traditional inhibitors to some extent. However, the light dependent nature may limit its application in deep tumors. The *in vivo* delivery and pharmacokinetics still need to be systematically evaluated, and tumor heterogeneity may affect the degradation efficiency. Therefore, more animal models and long-term safety studies are still needed before entering clinical application.

## 6. Limitations of LYTACs

LYTACs represent a promising strategy for targeted degradation of extracellular and membrane proteins. However, several key limitations may hinder their translational potential. First, LYTAC technology efficacy is highly dependent on the expression of lysosomal trafficking receptors. For example, an EGFR-targeting LYTAC achieved about 60%–70% degradation in HeLa cells, but this effect was significantly reduced upon CI-M6PR (IGF2R) knockdown, underscoring strict receptor dependence (Banik et al. 2020). Given the substantial variability of CI-M6PR expression across tissues, such heterogeneity may limit therapeutic applicability. Similarly, GalNAc-conjugated LYTACs selectively induced EGFR degradation in ASGPR positive hepatocyte derived cells, with minimal activity observed in ASGPR negative contexts (Ahn et al. 2021). Thus, receptor availability is a primary determinant of LYTAC efficacy. Second, receptor mediated endocytosis alone does not ensure efficient lysosomal degradation. Intracellular trafficking, including endosomal sorting, lysosomal delivery, and receptor recycling, can impose additional constraints. Notably, only partial EGFR degradation (about 50%–60%) was observed after 24 h of treatment, with near maximal effects requiring prolonged exposure, suggesting that post-endocytic processing is rate-limiting (Banik et al. 2020). Third, suboptimal pharmacokinetics and stability remain challenges. Many LYTACs rely on peptide or glycopeptide ligands (e.g. M6P) that are susceptible to enzymatic degradation. Although more stable analogs, such as M6Pn have been developed (Gary-Bobo et al. 2007), the large size and structural complexity of LYTACs may still limit tissue penetration and systemic exposure. Finally, LYTACs carry a risk of off-target toxicity. The IGF2R mutant exhibited significantly increased accumulation in the spleen in mouse models, suggesting a potential enhancement in its affinity for spleen-related targets or immune cells. However, it also showed notable distribution in bone as early as 4 h after administration (Prabaharan et al. 2023). Strategies such as tissue specific receptor targeting, conditional activation, and optimized linker design may mitigate these risks, but remain to be fully validated.

## 7. Conclusion and perspectives

LYTAC technology facilitates the selective degradation of extracellular proteins and membrane proteins whose functional domains reside in the extracellular space, thereby effectively circumventing the inherent constraints of PROTACs arising from their reliance on intracellular target engagement. For key membrane bound or secreted proteins, such as PD-L1 and CD24, which drive tumor immune evasion and therapeutic resistance, LYTACs direct these targets to the lysosomal degradation pathway, achieving functional clearance that is difficult to attain with conventional small-molecule or antibody-based therapies. Ongoing optimization of LYTACs, including multivalent ligand design, receptor-independent targeting strategies, and integration with nanotechnology has further improved degradation efficiency, *in vivo* stability and tissue distribution. By alleviating immunosuppression within the tumor microenvironment and synergizing with immunotherapies, LYTAC-mediated sustained target degradation holds significant promise for enhancing treatment sensitivity, overcoming resistance, and enabling more precise anticancer therapies.

Looking ahead, LYTAC technology is expected to advance rapidly through improved molecular design, delivery strategies, and mechanistic understanding. Next generation LYTACs will likely feature more stable linkers, modular architectures, and receptor independent uptake, broadening their applicability across tumor types. Early CI-M6PR dependent LYTACs typically employed highly polymerized multivalent M6P ligands conjugated to antibodies through flexible PEG linkers to enhance receptor binding. Yet excessively long or flexible PEG chains can reduce cellular uptake efficiency due to increased conformational entropy and a lower proportion of bioactive conformations. Future studies integrating structural biology and computational modeling to rationally optimize linker architecture may further stabilize ternary complexes and enhance endocytic trafficking, ultimately improving the degradation efficiency of LYTACs. Furthermore, targeting tumor specific endocytosis and using nanocarriers or tumor targeting ligands may further enhance selective delivery. Expanding LYTACs to degrade extracellular cytokines, matrix components, and soluble resistance factors offers additional therapeutic potential. Biomarker-guided patient selection and integration with nanotechnology or synthetic biology may improve pharmacokinetics and therapeutic control. Overall, continued refinement will help position lysosomal degradation as a promising strategy to overcome cancer drug resistance.

## Supplementary Material

RightsLink Reprintable License of Figure 5.pdf RightsLink Reprintable License of Figure 5.pdf

RightsLink Reprintable License of Figure 2.pdf RightsLink Reprintable License of Figure 2.pdf

RightsLink Reprintable License of Figure 4.pdf RightsLink Reprintable License of Figure 4.pdf

RightsLink Reprintable License of Figure 3.pdf RightsLink Reprintable License of Figure 3.pdf

RightsLink Reprintable License of Figure 6.pdf RightsLink Reprintable License of Figure 6.pdf

## Data Availability

Data sharing is not applicable to this article as no new data were created or analyzed in this study.
