# Supplementary material for: Lysosome-directed targeted protein degradation technologies for overcoming cancer drug resistance: mechanisms, design principles, and therapeutic opportunities
Source: Drug Deliv. 2026 May 27;33(1):2679844. doi: 10.1080/10717544.2026.2679844 (PMC13220583; doi:10.1080/10717544.2026.2679844)
Supplement: RightsLink Reprintable License of Figure 5.pdf [file IDRD_A_2679844_SM3319.pdf]

CCC Marketplace

Order Confirmation

Thank you, your order has been placed. An email confirmation has been sent to you. Your order license details and printable licenses will be available within 24 hours. Please access Manage Account for final order details.

This is not an invoice. Please go to manage account to access your order history and invoices.

CUSTOMER INFORMATION

Payment by credit card: Your order will be finalized and your card will be charged within 24 hours.

Billing Address

Mr. Weidong Fei
Xueshi Road No. 1, Shangcheng District
Hangzhou, Zhejiang 310006
China

+86 13867419241
feiweidong@zju.edu.cn

Customer Location

Mr. Weidong Fei
Xueshi Road No. 1, Shangcheng District
Hangzhou, Zhejiang 310006
China

PO Number (optional)

N/A

Payment options

CC ending in 9107

PENDING ORDER CONFIRMATION

Confirmation Number: Pending

Order Date: 16-Mar-2026

1. Chemical communications

219.50 USD

Article: Targeted Degradation of ABCG2 for Reversing Multidrug Resistance by Hypervalent Bispecific Gold Nanoparticle-Anchored Aptamer Chimeras

**Order License ID**	Pending	**Publisher**	ROYAL SOCIETY OF CHEMISTRY
**ISSN**	1364-548X		
**Type of Use**	Republish in a journal under STM Guidelines	**Portion**	Chart/graph/table/figure

LICENSED CONTENT

**Publication Title**	Chemical communications	**Publication Type**	e-Journal
**Article Title**	Targeted Degradation of ABCG2 for Reversing Multidrug Resistance by Hypervalent Bispecific Gold Nanoparticle-Anchored Aptamer Chimeras	**Start Page**	3118
		**End Page**	3121
		**Issue**	21
		**Volume**	59
**Author / Editor**	Royal Society of Chemistry (Great Britain)	**URL**	http://pubs.rsc.org/en/journals/journalissues/cc
**Date**	01/01/1996		
**Language**	English		
**Country**	United Kingdom of Great Britain and Northern Ireland		
**Rightsholder**	Royal Society of Chemistry		

REQUEST DETAILS

**Portion Type**	Chart/graph/table/figure	**Rights Requested**	Main product
**Number of Charts / Graphs / Tables / Figures Requested**	4	**Distribution**	Worldwide
		**Translation**	Original language of publication
**Format (select all that apply)**	Print, Electronic	**Copies for the Disabled?**	No
**Who Will Republish the Content?**	Publisher, STM	**Minor Editing Privileges?**	No
**STM Signatories**	Taylor & Francis (Journals)	**Incidental Promotional Use?**	No
**Duration of Use**	Life of current edition	**Currency**	USD
**Lifetime Unit Quantity**	Up to 499		

NEW WORK DETAILS

**Title**	Lysosome-directed targeted protein degradation technologies for overcoming cancer drug resistance: mechanisms, design principles, and therapeutic opportunities
**Author**	Huixin Mao , Ying Zhang, Mingqi Liu, Yao Yao, Yujie Peng, Xuwei Zhou, Hao Huang, Weidong Fei, Caihong Zheng , Yiqing Ye
**Publisher**	Taylor & Francis (Journals)
**Publisher Imprint**	N/A
**Expected Publication Date**	2026-05-13
**Available in the Following Markets**	Worldwide
**Expected Size of the New Work (number of pages)**	30
**Proposed Price**	N/A
**Standard Identifier**	N/A

ADDITIONAL DETAILS

**Order Reference Number**	N/A
**The Requesting Person / Organization to Appear on the License**	Weidong Fei/Women’s Hospital, Zhejiang University School of Medicine

REQUESTED CONTENT DETAILS

**Title, Description or Numeric Reference of the Portion(s)**	Scheme 1 ,Figure 2 ,Figure 3, and Figure 4
**Editor of Portion(s)**	Lu, Weihua; Chen, Jingran; Guo, Zhanchen; Ma, Yanyan; Gu, Zikuan; Liu, Zhen
**Volume / Edition**	59
**Page or Page Range of Portion**	3118-3121
**Title of the Article / Chapter the Portion Is From**	Targeted Degradation of ABCG2 for Reversing Multidrug Resistance by Hypervalent Bispecific Gold Nanoparticle-Anchored Aptamer Chimeras
**Author of Portion(s)**	Lu, Weihua; Chen, Jingran; Guo, Zhanchen; Ma, Yanyan; Gu, Zikuan; Liu, Zhen
**Issue, if Republishing an Article From a Serial**	21
**Publication Date of Portion**	2023-03-09

Total Items: 1

Total Due: 219.50 USD

Accepted: Marketplace Permissions General Terms and Conditions and any applicable Publisher Terms and Conditions
