# Supplementary material for: Lysosome-directed targeted protein degradation technologies for overcoming cancer drug resistance: mechanisms, design principles, and therapeutic opportunities
Source: Drug Deliv. 2026 May 27;33(1):2679844. doi: 10.1080/10717544.2026.2679844 (PMC13220583; doi:10.1080/10717544.2026.2679844)
Supplement: RightsLink Reprintable License of Figure 2.pdf [file IDRD_A_2679844_SM3320.pdf]

CCC | Marketplace

Sign in/Register

All types | Nano-LYTACs for Degradation of Membrane Proteins and Inhibition of CD24/Siglec-10 Signaling Pathway

Advanced Search | Search Tips

Filter your results:

No filters are available

0 publications and 2 articles/chapters matched your search term(s)

Hide filters

Article/Chapter Results

Sort by Relevance

Nano-LYTACs for Degradation of Membrane Proteins and Inhibition of CD24/Siglec-10 Signaling Pathway.

Wang, Kun; Yu, Albert; Liu, Kewei; Feng, Chunyan; Hou, ...More *Advanced Science*, 15 Sep 2023, Vol. 10, Issue 26,

ISSN: 21983844

DOI: 10.1002/advs.202305364

PMID: 37712353

PMCID: PMC10502625

Publisher: Wiley

Language: English

Country: Germany

URL: https://onlinelibrary.wiley.com/toc/21983844/10/26

Request Reprints/ePrints | Request Single Copy | **Open Access** - Creative Commons CC BY 4.0
