# Supplementary material for: Lysosome-directed targeted protein degradation technologies for overcoming cancer drug resistance: mechanisms, design principles, and therapeutic opportunities
Source: Drug Deliv. 2026 May 27;33(1):2679844. doi: 10.1080/10717544.2026.2679844 (PMC13220583; doi:10.1080/10717544.2026.2679844)
Supplement: RightsLink Reprintable License of Figure 4.pdf [file IDRD_A_2679844_SM3321.pdf]

CCC | Marketplace

HM

All types | A Self-Assembling LYTAC Mediates CTGF Degradation and Remodels Inflammatory Tumor Microenvironment

Advanced Search | Search Tips

Filter your results:

No filters are available

0 publications and 1 articles/chapters matched your search term(s)

Hide filters

Article/Chapter Results

Sort by Relevance

A Self - Assembling LYTAC Mediates CTGF Degradation and Remodels Inflammatory Tumor Microenvironment for Triple - Negative Breast Cancer Therapy

Lin, Jia - Yi; Wu, Ye; Liang, Xiao - Hui; Tang, Min; Sun, ...More *Advanced Science*, 20 Jun 2025, Vol. 12, Issue 23, pages n/a - n/a

ISSN: 21983844
DOI: 10.1002/advs.202500311
PMID: 40349150
PMCID: PMC12199414

Publisher: Wiley
Language: English
Country: Germany
URL: https://advanced.onlinelibrary.wiley.com/toc/21983844/12/23

Details >

Request Reprints/ePrints | Request Single Copy | Open Access - Creative Commons CC BY 3.0
