# Supplementary material for: Lysosome-directed targeted protein degradation technologies for overcoming cancer drug resistance: mechanisms, design principles, and therapeutic opportunities
Source: Drug Deliv. 2026 May 27;33(1):2679844. doi: 10.1080/10717544.2026.2679844 (PMC13220583; doi:10.1080/10717544.2026.2679844)
Supplement: RightsLink Reprintable License of Figure 6.pdf [file IDRD_A_2679844_SM3323.pdf]

AMERICAN CHEMICAL SOCIETY LICENSE
TERMS AND CONDITIONS

Feb 26, 2026

This Agreement between Department of Pharmaceutics, Zhejiang University, Caihong Zheng ("You") and American Chemical Society ("American Chemical Society") consists of your license details and the terms and conditions provided by American Chemical Society and Copyright Clearance Center.

License Number	6216350722184
License date	Feb 26, 2026
Licensed Content Publisher	American Chemical Society
Licensed Content Publication	Journal of the American Chemical Society
Licensed Content Title	Phototriggered LYTAC: Photoactive Bispecific Aptamer Chimera Enhances Targeted Degradation of Membrane Protein through Regulating Cell Autophagy
Licensed Content Author	Rongjun Zhang, Changjie Yang, Xiaobo Gao, et al
Licensed Content Date	Jun 1, 2025
Licensed Content Volume	147
Licensed Content Issue	24
Volume number	147

Issue number	24
Type of Use	Journal
Requestor type	Author's Employer
Format	Electronic
Portion	Table/Figure/Micrograph
Number of Table/Figure/Micrographs	4
Title of new article	Lysosome-directed targeted protein degradation technologies for overcoming cancer drug resistance: mechanisms, design principles, and therapeutic opportunities
Lead author	Caihong Zheng, Yiqing Ye,Weidong Fei
Title of targeted journal	Drug Drlivery
Publisher	Taylor & Francis
Expected publication date	Mar 2026
Portions	Scheme 1, Figure 3, Figure 5 and Figure 8
The Requesting Person / Organization to Appear on the License	Department of Pharmaceutics, Zhejiang University, Caihong Zheng
Requestor Location	huixin mao<br>Department of Pharmaceutics

	Hangzhou, Zhejiang 310058<br>China
Payment Type	Invoice
Email Address	22519130@zju.edu.cn
Billing Address	zhejiang university<br>Department of Pharmaceutics<br><br>Hangzhou, China 310058
Total	0.00 USD

Terms and Conditions

ACS / RIGHTSLINK TERMS & CONDITIONS

INTRODUCTION
The publisher for this copyrighted material is the American Chemical Society. By clicking "accept" in connection with completing this licensing transaction, you agree that the following terms and conditions apply to this transaction (along with the Billing and Payment terms and conditions established by Copyright Clearance Center, Inc. ("CCC"), at the time that you opened your RightsLink account and that are available at any time at <http://myaccount.copyright.com>).

LIMITED LICENSE
Publisher hereby grants to you a non-exclusive license to use this material. Licenses are for one-time use only with a maximum distribution equal to the number that you identified in the licensing process. Note that if credit is given to another source for the material you requested from RightsLink, permission must be obtained from that source and not the ACS.

GEOGRAPHIC RIGHTS: SCOPE
Licenses may be exercised anywhere in the world.

RESERVATION OF RIGHTS
Publisher reserves all rights not specifically granted in the combination of (i) the license details provided by you and accepted in the course of this licensing

transaction, (ii) these terms and conditions and (iii) CCC's Billing and Payment terms and conditions.

PORTION RIGHTS STATEMENT: DISCLAIMER
If you seek to reuse a portion from an ACS publication, it is your responsibility to examine each portion as published to determine whether a credit to, or copyright notice of, a third party owner was published adjacent to the item. You may only obtain permission via RightsLink to use material owned by ACS. Permission to use any material published in an ACS publication, journal, or article which is reprinted with permission of a third party must be obtained from the third party owner. ACS disclaims any responsibility for any use you make of items owned by third parties without their permission.

REVOCATION
The American Chemical Society reserves the right to revoke a license for any reason, including but not limited to advertising and promotional uses of ACS content, third party usage, and incorrect figure source attribution.

LICENSE CONTINGENT ON PAYMENT
While you may exercise the rights licensed immediately upon issuance of the license at the end of the licensing process for the transaction, provided that you have disclosed complete and accurate details of your proposed use, no license is finally effective unless and until full payment is received from you (by CCC) as provided in CCC's Billing and Payment terms and conditions. If full payment is not received on a timely basis, then any license preliminarily granted shall be deemed automatically revoked and shall be void as if never granted. Further, in the event that you breach any of these terms and conditions or any of CCC's Billing and Payment terms and conditions, the license is automatically revoked and shall be void as if never granted. Use of materials as described in a revoked license, as well as any use of the materials beyond the scope of an unrevoked license, may constitute copyright infringement and publisher reserves the right to take any and all action to protect its copyright in the materials.

COPYRIGHT NOTICE: DISCLAIMER
You must include the following copyright and permission notice in connection with any reproduction of the licensed material: "Reprinted ("Adapted" or "in part") with permission from REFERENCE CITATION. Copyright YEAR American Chemical Society."

WARRANTIES: NONE
Publisher makes no representations or warranties with respect to the licensed material.

INDEMNITY
You hereby indemnify and agree to hold harmless publisher and CCC, and their respective officers, directors, employees and agents, from and against any and all claims arising out of your use of the licensed material other than as specifically authorized pursuant to this license.

NO TRANSFER OF LICENSE
This license is personal to you or your publisher and may not be sublicensed, assigned, or transferred by you to any other person without publisher's written permission.

NO AMENDMENT EXCEPT IN WRITING
This license may not be amended except in a writing signed by both parties (or, in the case of publisher, by CCC on publisher's behalf).

OBJECTION TO CONTRARY TERMS
Publisher hereby objects to any terms contained in any purchase order, acknowledgment, check endorsement or other writing prepared by you, which terms are inconsistent with these terms and conditions or CCC's Billing and Payment terms and conditions. These terms and conditions, together with CCC's Billing and Payment terms and conditions (which are incorporated herein), comprise the entire agreement between you and publisher (and CCC) concerning this licensing transaction. In the event of any conflict between your obligations established by these terms and conditions and those established by CCC's Billing and Payment terms and conditions, these terms and conditions shall control.

JURISDICTION
This license transaction shall be governed by and construed in accordance with the laws of the District of Columbia. You hereby agree to submit to the jurisdiction of the courts located in the District of Columbia for purposes of resolving any disputes that may arise in connection with this licensing transaction.

Other conditions:

v1.3

Questions? customercare@copyright.com.
